# OIP5-AS1 contributes to tumorigenesis in hepatocellular carcinoma by miR-300/YY1-activated WNT pathway

**DOI:** 10.1186/s12935-020-01467-6

**Published:** 2020-09-09

**Authors:** Yu Wang, Lei Dou, Yun Qin, Huiyuan Yang, Peng Yan

**Affiliations:** 1 Hepatic Surgery Center, Tongji Hospital, Tongji Medical College, Huazhong University of Science and Technology, Wuhan, 430030 Hubei China; 2 Hubei Province for the Clinical Medicine Research Center of Hepatic Surgery, Wuhan, 430030 Hubei China; 3 Key Laboratory of Organ Transplantation, Ministry of Education and Ministry of Public Health, Wuhan, 430030 Hubei China; 4 Department of Geratology, Tongji Hospital, Tongji Medical College, Huazhong University of Science and Technology, Wuhan, 430030 Hubei China; 5 Department of Hematology, Tongji Hospital of Tongji Medical College, Huazhong University of Science and Technology, 1095 Jie-Fang Avenue, Wuhan, 430030 Hubei China; 6 Department of Cancer Center, Tongji Hospital, Tongji Medical College, Huazhong University of Science and Technology, Wuhan, 430030 Hubei China

**Keywords:** OIP5-AS1, miR-300, YY1, WNT pathway, Hepatocellular carcinoma

## Abstract

**Background:**

It has reported that long non-coding RNAs (lncRNAs) exerted regulatory functions by targeting specific genes through a competing endogenous RNA (ceRNA) pathway. LncRNA OIP5-AS1 has been identified as a tumor-enhancer in several tumor types. Nonetheless, its molecular mechanism in HCC remains to be masked.

**Aim of the study:**

This study was aimed at exploring whether and how OIP5-AS1 exert functions in HCC.

**Methods:**

qRT-PCR and western blot were employed for detecting gene expression. CCK-8, colony formation and EdU assays were implemented to evaluate the proliferative ability of HCC cells. Caspase-3 activity and flow cytometry analyses were implemented to determine cell apoptosis and cell cycle distribution. RNA pull down, ChIP, RIP and luciferase reporter assays explored the interplays between molecules.

**Results:**

YY1 was upregulated in HCC cells, and silenced YY1 restrained HCC cell proliferation in vitro and hampered tumor growth in vivo. Later, we discovered that miR-300 could regulate WNT pathway via targeting YY1. Furthermore, OIP5-AS1 was identified as the sponge of miR-300 and promoted cell growth in HCC. Importantly, YY1 transcriptionally activate OIP5-AS1 in turn. Rescue experiments indicated that miR-300 inhibition or YY1 overexpression abrogated the inhibitive effect of OIP5-AS1 silencing on the malignant growth of HCC cells.

**Conclusions:**

OIP5-AS1/miR-300/YY1 feedback loop facilitates cell growth in HCC by activating WNT pathway. 
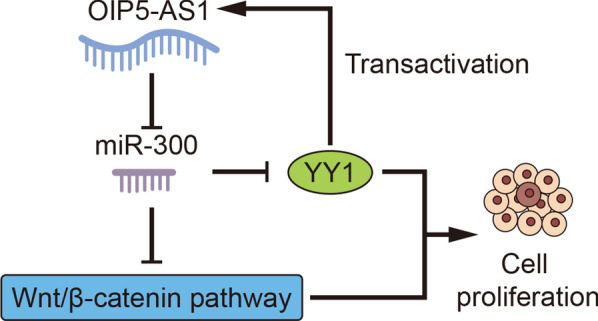

## Background

As the most frequent subtype of liver cancer [1], hepatocellular carcinoma (HCC) ranks second among the cancers contributing to death globally [2]. Recent years, the pre-clinical treatment strategies have been gradually reported [3, 4]. Despite great improvement in interventional therapy has been made for HCC, the clinical effect remains disappointed due to the difficulty in early diagnosis of HCC patients [5]. Thus, the imperious demand for novel biomarkers in HCC needs to be met.

Long non-coding RNAs (lncRNAs), greater than 200 nucleotides in length, have limitations in protein-coding capacity [6]. Current reports have disclosed the critical implication of lncRNAs in physiological and pathological, including organismal viability, immunity, tumorigenesis, organ development and tumor progression [7–9]. In addition, the roles that lncRNAs served in biological processes was reported via the regulation of gene transcription, mRNA processing, protein activity or nuclear domains organization [10, 11]. In recent decades, it has been identified that dysregulation of lncRNAs is closely connected with the development of human cancers [12]. For example, lncRNA XIST enhances thyroid cancer cell growth by regulating miR-34a/MET-PI3K-AKT signaling [13]. LINC01296 contributes to the progression of colorectal cancer by modulating miR-26a/GALNT3 axis and PI3K/AKT pathway [14]. LINC00511 sponges miR-185-3p in breast cancer and facilitates tumorigenesis and stemness via inducing E2F1/Nanog axis [15]. LncRNA OIP5-AS1 has been reported to exert its oncogenic functions in osteosarcoma [16], melanoma [17] and bladder cancer [18]. However, whether and how OIP5-AS1 works in HCC have not been studied.

MicroRNAs (miRNAs) are known as tumor suppressors or promoters by targeting their downstream mRNAs [19, 20]. In recent years, the role of miRNAs in ceRNA network has been unveiled [21, 22]. MiR-300 has been proved as a tumor inhibitor in various human cancers [23, 24]. However, it is unclear whether OIP5-AS1 and miR-300 could interact with each other. Here, we aimed at figuring out the functions of OIP5-AS1in HCC cell growth, as well as the downstream molecules of OIP5-AS1 in modulating HCC.

## Methods

### Cell culture and processing

Human liver epithelium cell line (THLE-3) and HCC cell lines (HepG2, LM3, Hep3B, Huh7 and MHCC97H) utilized in our study were purchased from American Type Culture Collection (Manassas, VA, USA). Cells were then cultivated under 5% CO_2_ at 37 °C in RPMI 1640 medium (Invitrogen, Carlsbad, CA, USA) with 10% fetal bovine serum (FBS; Gibco, Grand Island, NY, USA) and 100 mg/ml penicillin/streptomycin (P/S; Gibco).

### Quantitative real-time polymerase chain reaction (qRT-PCR)

By utilizing Trizol reagent (TaKaRa, Tokyo, Japan), the total RNA was extracted from HepG2 and MHCC97H cells as requested of protocol. Following identifying RNA concentration and quality, cDNA was obtained by reverse transcription with Primescipt RT reagent kit (TaKaRa). Afterwards, qRT-PCR was operated via SYBR Premix Ex Taq II kit (TaKaRa). GAPDH was the endogenous control. Relative expression was tested based on 2^−ΔΔCt^ method. PCR primers were provided in Additional file 1: Table S1.

### Cell transfection

The plasmids used in our study were acquired commercially from RiboBio (Guangzhou, China), including short hairpin RNAs specific to OIP5-AS1 and YY1, miR-300 mimics and YY1 overexpression plasmid, as well as their negative controls (non-specific shRNA, NC mimics and empty pcDNA3.1 vector). Transfection of indicated plasmids into HepG2 and MHCC97H cells was realized under the help of Lipofectamine2000 (Invitrogen).

### Cell counting kit-8 (CCK-8) assay

HCC or THLE-3 cells were incubated at 37 °C and then reaped at 24, 48, 72 and 96 h, followed by the addition of CCK-8 solution (Dojindo Laboratories, Kumamoto, Japan). Four hours later, absorbance was assayed at 450 nm by microplate reader.

### Colony formation assay

The transfected HCC or THLE-3 cells were planted into 6-well plates, followed by 14-day cultivation at 37 °C under a condition with 5% CO_2_. Then, the medium was removed and the plates were scoured in phosphate-buffered saline (PBS; Invitrogen) for 2 times. After being fixed, colonies were subjected to staining via 0.1% crystal violet solution (1 mL). At length, colonies number was counted manually.

### EdU incorporation assay

After transfection, HCC or THLE-3 cells were planted in 24-well plates with sterile coverslips and then cell proliferation was assessed via EdU kit (RiboBio) in accordance with the specification. Cell nuclei were processed consecutively with EdU and DAPI (4′,6-diamidino-2-phenylindole; Beyotime, Shanghai, China). Finally, images were captured with laser confocal microscopy (Olympus, Tokyo, Japan).

### Caspase-3 activity test

A caspase-3 activity kit (Solarbio, Beijing, China) was utilized for measurement of caspase-3 activity in cell lysates as per the standard instruction. Total protein from indicated cells was mixed with caspase-3 substrate and reaction buffer. The absorbance was assayed by a microplate reader at 405 nm after 4 h of incubation at 37 °C.

### Western blotting

Lysates of HepG2 and MHCC97H cells were acquired by use of RIPA buffer (Thermo Fisher Scientific, MA, USA), followed by proteins in the lysates were subjected to BCA kit (Thermo Fisher Scientific) for concentration detection. Samples were then separated on 10% SDS-PAGE gel and subjected to transferring onto polyvinylidene difluoride (PVDF) membrane. After being blocked, membranes were cultured at 4 °C with primary antibodies overnight and then with horseradish peroxidase-conjugated secondary antibody. Antibodies were acquired from Abcam (Cambridge, MA, USA) as follows: anti-Bcl-2 (ab692), anti-Bax (ab53154), anti-β-catenin (ab32572), anti-Axin2 (ab32197), anti-phosphorylated β-catenin (ab75777), anti-cyclin D1 (ab40754), anti-c-myc (ab39688), anti-Histone H3 (ab1791), anti-YY1 (ab109228) and anti-GAPDH (ab37168). Protein signals were detected by ECL method (Thermo Fisher Scientific).

### TOP/FOP flash reporter assay

The activity of WNT pathway was assessed by performing TOP/FOP Flash assay. Briefly, reporters containing TOP Flash or FOP Flash were synthesized and obtained from Addgene. Cells were placed in a 96-well plate, followed by co-transfection with indicated plasmids (GenePharma) and TOP Flash or FOP Flash vector (Promega). One day later, Promega Dual-Luciferase Reporter Assay System was applied for evaluating the luciferase activity, with activity of Renilla as normalizing control.

### Immunofluorescence (IF) assay

The distribution of β-catenin was measured as previously described [25]. After transfections, cells were fixated in 4% paraformaldehyde for 15 min and then processed with 0.1% Triton X-100 (ST795, Beyotime Institute of Biotechnology). After being sealed via goat serum, the slides were subjected to processing at 4 °C with anti-β-catenin (51067-2-AP; 1:200 dilution, Proteintech) overnight, and at 25 °C with secondary antibody (A0516; 1:200 dilution, Beyotime Institute of Biotechnology). Following nuclei-staining via DAPI, images were obtained using a microscope (Olumpus, Japan).

### Cell aggregation assay

Cell aggregation assay was conducted in accordance with previous report [26].

### In vivo experiment

Male 6-8 weeks BALB/C nude mice were bought from Shanghai SIPPR-BK Laboratory (Shanghai, China) and kept in micro-isolator cages. All the animal assays were conducted with the consent of Ethics Institution of Tongji Hospital (approval number: 81600503). Serum-free culture medium with 5 × 10^6^ MHCC97H cells transfected with sh-YY1#1 or sh-NC (control or miR-300 agomir; sh-NC or sh-OIP5-AS1#1) were subcutaneously injected into mice. The volume of tumors was tested every 4 days. 28 days later, the mice were sacrificed and the tumors were weighed and collected. After that, tumors were fixed, paraffin-embedded and sliced for hematoxylin–eosin (HE) or immunohistochemical (IHC) staining in line with former protocols [27].

### RIP assay

Magna RIP RNA-Binding Protein Immunoprecipitation Kit (Millipore) was applied for conducting this assay in HepG2 or MHCC97H cells. After lysing with RIP buffer, the acquired cell lysates were processed with ProteinA/G magnetic beads conjugated with anti-Ago2 (Abcam) or anti-IgG (Abcam). The enrichment of RNAs in immunoprecipitates were estimated by qRT-PCR.

### RNA pull-down

The sequences of miR-NC, miR-300-WT and miR-300-Mut were labeled with biotin by GenePharma (Shanghai, China) for acquiring Bio-miR-NC, Bio-miR-300-WT and Bio-miR-300-Mut, which were then subjected to incubation with cell lysates and Dyna-beads M-280 Streptavidin (Invitrogen) for overnight. The pulled-down RNAs were tested via qRT-PCR.

### Luciferase reporter assay

The pmirGLO-YY1-WT (wild-type) or pmirGLO-OIP5-AS1-WT was formed via cloning YY1 3′UTR or full-length OIP5-AS1 with the predictive binding sites of miR-300 into pmirGLO Dual-Luciferase miRNA Target Expression Vector (Promega Corporation, Fitchburg, WI, USA). Meanwhile, the pmirGLO-YY1-Mut (mutation) or pmirGLO-OIP5-AS1-Mut vector was similarly generated. After that, above reporters were individually co-transfected into HepG2 or MHCC97H cells with miR-300 mimics or NC mimics. The OIP5-AS1 promoter with wild or mutant type of YY1 binding sites was separately inserted into pGL3 luciferase reporter vector (Promega Corporation). Two days later, the luciferase activity was tested by use of Dual-Luciferase Reporter Assay System (Promega).

### Chromatin immunoprecipitation (ChIP)

ChIP assay was conducted with the Magna ChIP Kit (Millipore). In brief, following sonication of cross-linked chromatin to the fragments, cell lysates were processed with anti-YY1 or anti-IgG overnight. Thereafter, the precipitated DNA fragments were estimated via qRT-PCR.

### Nucleus-cytoplasm separation assay

HepG2 and MHCC97H cells were rinsed twice in PBS. Cell lysates from cell fractionation buffer were centrifuged to isolate nucleus and cytoplasm. The supernatant was seen as cytoplasmic fraction. The remaining lysates was washed, centrifuged and treated with cell disruption buffer. The relative levels of β-catenin in nuclear or cytoplasmic fractions were detected by qRT-PCR, with Histone H3 as the nuclear control and GAPDH as the cytoplasmic control.

### Statistical analysis

All experimental data obtained from no less than 2 replicates were exhibited as mean ± standard deviation (S.D.) after evaluating via GraphPad PRISM 6 (GraphPad, San Diego, CA, USA). Variance between groups was assayed by Student’s t test or one-way ANOVA, as appropriate. The threshold of statistical significance was deemed a P < 0.05.

## Results

### YY1 is upregulated and facilitates tumorigenesis in HCC

**Fig. 1:**
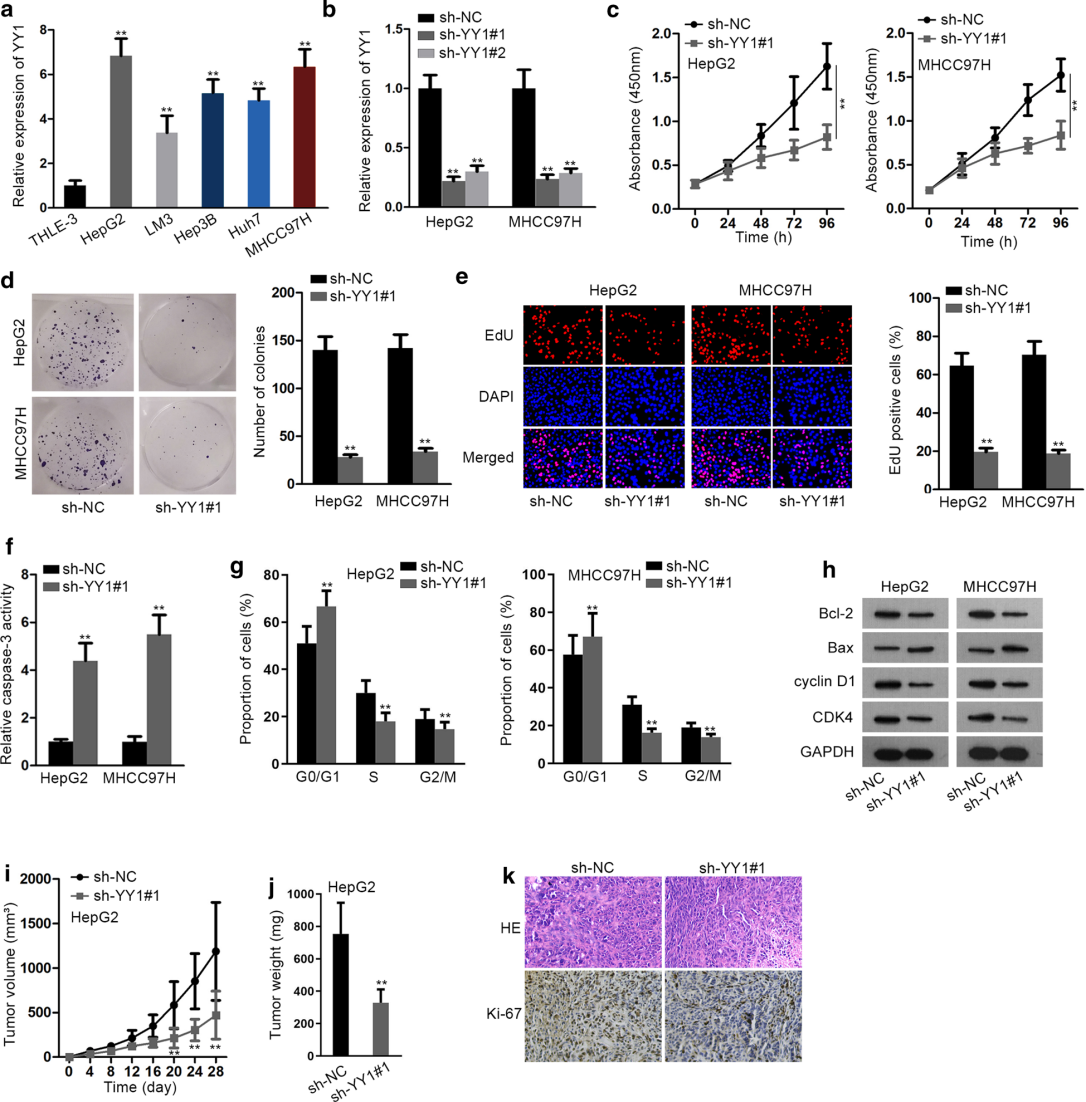
YY1 is upregulated in HCC and facilitates HCC tumor growth. **a** YY1 expression in five HCC cell lines and one human liver epithelium cell line (THLE-3) was detected by qRT-PCR. **b** HepG2 and MHCC97H cells that separately transfected with sh-NC or sh-YY1#1/2 were analyzed by qRT-PCR for YY1 expression. **c**–**e** The proliferation was assessed by CCK-8, colony formation and EdU assays in sh-YY1#1 transfected HepG2 and MHCC97H cells. **f** Caspase-3 activity in YY1-downregulated HCC cells was measured. **g** Apoptosis- and cell cycle-related proteins were detected in HCC cells transfected with sh-NC or sh-YY1#1 by western blot analysis. **h** Cell cycle distribution was assessed by flow cytometry analysis in HCC cells after YY1 silence. **i**, **j** The growth curve and weight of tumors removed from the mice injected with sh-NC or sh-YY1#1 transfected MHCC97H cells were tested. **k** IHC assay measured the Ki67 staining in tumors from indicated groups. **P < 0.01

At first, we determined that YY1 was upregulated in HCC cells (HepG2, LM3, Hep3B, Huh7 and MHCC97H) in comparison to human normal THLE-3 cells (Fig. 1a). To probe the function of YY1 in HCC, special shRNAs against YY1 (sh-YY1#1 or sh-YY1#2) were transfected into HepG2 and MHCC97H cells to knock down YY1 expression. As a result, both sh-YY1#1 and sh-YY1#2 silenced YY1 expression, and sh-YY1#1 was used for the following experiments due to higher interfering efficiency when the transfection efficiencies were almost the same (Fig. 1b and Additional file 2: Fig. S1A). Later, we revealed that YY1 deficiency evidently restrained the proliferation of HCC cells (Fig. 1c–e). Then, caspase-3 activity assay was performed to evaluate cell apoptosis in HCC. Data depicted that caspase-3 activity was obviously increased in HCC cells with the transfection of sh-YY1 (Fig. 1f). Furthermore, we observed an evident arrest of cell cycle at G0/G1 phase when inhibiting YY1 (Fig. 1g). Further, western blot analyzed the levels of proteins associated with apoptosis (Bcl-2 and Bax) and cell cycle (cyclin D1 and CDK4), delineating the reduced Bcl-2, cyclin D1 and CDK4 levels and increased Bax level in HCC cells in response to YY1 suppression (Fig. 1h and Additional file 2: Fig. S1B). More importantly, the outcomes of in vivo experiments unveiled that the tumors grew slower and their weights were lighter in sh-YY1#1 group than those in sh-NC group (Fig. 1i, j). In addition, we also observed the lessened Ki67 positivity in tumors from YY1-depleted group (Fig. 1k). To determine whether YY1 also served an oncogenic impact on normal hepatocytes, we then overexpressed YY1 in THLE-3 cells and detected the functional changes. Fortunately, results indicated that although the transfection efficiencies were similar, YY1 expression was apparently boosted in THLE-3 cells transfected with pcDNA3.1-YY1 (Additional file 2: Fig. S1C, D). After that, gain-of-function assays revealed that overexpression of YY1 had no significant effects on the proliferation, apoptosis and cell cycle distribution of THLE-3 cells (Additional file 2: Fig. S1E–J), proving the specific contribution of YY1 to oncogenic phenotypes of malignant cells. Based on previous studies, YY1 can activate WNT pathway. Here, we explored the role of YY1 in the transcription of WNT pathway core factors. The results indicated that YY1 silencing reduced the transcription activity of CTNNB1 (Additional file 3: Fig. S2A). To further validate the activating influence of YY1 on WNT pathway, we evaluated the changes of WNT activity in HCC cells with different YY1 expressions. Prior to that, HepG2 and MHCC97H cells were transfected with different doses of pcDNA3.1/YY1. As a result, YY1 level was gradually elevated with the increase dose of pcDNA3.1/YY1, while the transfection efficiencies showed no apparent differences between the four doses (Additional file 3: Fig. S2B, C). Of note, we determined that overexpression of YY1 enhanced the luciferase activity of TOP flash but not FOP, and such enhancement was fortified in a dose-dependent manner (Additional file 3: Fig. S2D). Above findings indicated that YY1 is upregulated in HCC and promotes HCC tumorigenesis.

### MiR-300 targets to YY1 and inactivates WNT pathway in HCC

**Fig. 2:**
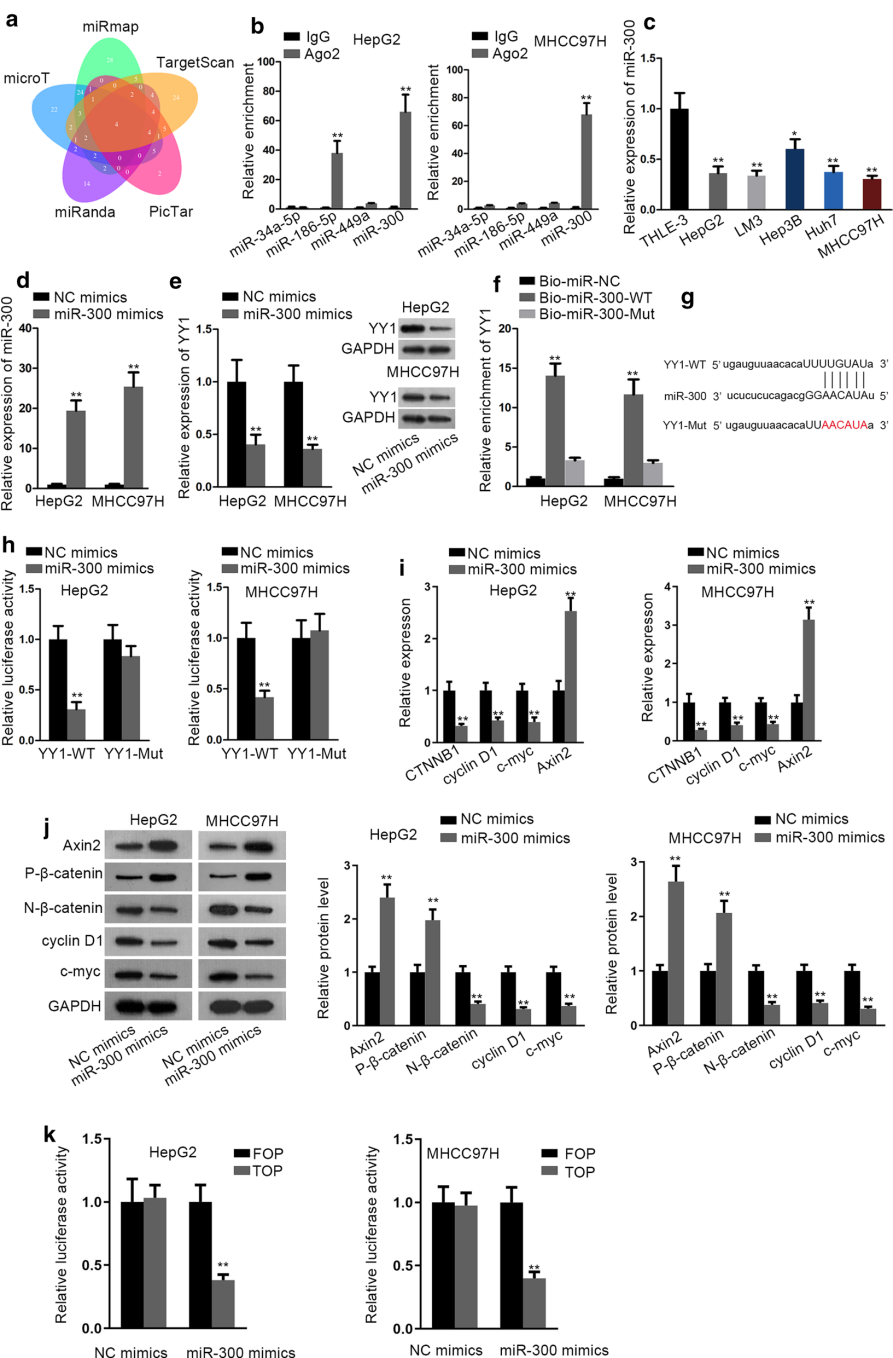
MiR-300 targets to YY1 and inactivates WNT pathway in HCC. **a** The upstream miRNAs of YY1 were predicted by five programs in starBase (including miRmap, TargetScan, PicTar, miRanda and microT), and the prediction results were showed by Venn. **b** RIP assay plus qRT-PCR analyzed the enrichment of candidate miRNAs in RISCs in HepG2 and MHCC97H cells. **c** MiR-300 expression in five HCC cell lines and one human liver epithelium was evaluated via qRT-PCR. **d** qRT-PCR detected miR-300 expression in HepG2 and MHCC97H cells transfected miR-300 mimics or NC mimics. **e** YY1 mRNA and protein levels were measured by qRT-PCR and western blot in HepG2 and MHCC97H cells with the transfection of miR-300 mimics or NC mimics. **f** RNA pull down assay helped to determine the interaction of miR-300 with YY1. **g** StarBase predicted the binding sequence of miR-300 in YY1 3′UTR. **h** Luciferase reporter assay was performed to verify the interaction between miR-300 and YY1. **i**, **j** The effect of miR-300 mimics on the expression of factors related to WNT pathway was detected by qRT-PCR and western blot analyses. **k** TOP/FOP flash assay determined the activity of WNT pathway in cells transfected with NC mimics or mi-300 mimics. *P < 0.05, **P < 0.01

Next, we attempted to look for the upstream factors of YY1 in HCC. In this regard, five databases (including miRmap, TargetScan, PicTar, miRanda and microT) were searched, and four candidates (miR-34a-5p, miR-186-5p, miR-449a and miR-300) were obtained (Fig. 2a). Further, RIP assay showed that among the four candidates, only miR-300 possessed obvious enrichment in Ago2-assambled RNA-induced silencing complexes (RISCs) in both the two HCC cells (Fig. 2b). Therefore, we speculated that miR-300 potentially bound to YY1 in HCC. Then, miR-300 level was found to be lessened in HCC cells (Fig. 2c). Later, miR-300 was overexpressed with the transfection of miR-300 mimics for further study (Fig. 2d). Furthermore, downregulated expression of YY1 mRNA and protein was detected in HCC cells in face of miR-300 upregulation (Fig. 2e and Additional file 3: Fig. S2E). Through RNA pull down experiment, we found the remarkable enrichment of YY1 in the beads conjugated with Bio-miR-300-WT (Fig. 2f). Then, the complementary sequence of YY1 for miR-300 was illustrated in Fig. 2g. In addition, luciferase reporter assay displayed that upregulating miR-300 had a specifically suppressive impact on the luciferase activity of YY1-WT (Fig. 2h). MiR-300 was uncovered to regulate WNT pathway in pancreatic cancer [28]. Here, we found that mRNA levels of CTNNB1, cyclin D1 and c-myc, were all decreased in HCC cells after transfecting with miR-300 mimics, whereas that of Axin2 were increased under the same condition (Fig. 2i). Consistently, the levels of non-phosphorylated β-catenin (N-β-catenin: namely total β-catenin), cyclin D1 and c-myc proteins were also reduced, whereas that of Axin2 and phosphorylated β-catenin (P-β-catenin) were augmented in miR-300-elevated HCC cells (Fig. 2j). Besides, TOP/FOP flash examination further revealed the inactivation of WNT pathway under miR-300 upregulation (Fig. 2k). More importantly, enhanced miR-300 suppressed the nuclear staining of β-catenin in HCC cells (Additional file 3: Fig. S2F). Above findings suggested that miR-300 targets to YY1 and inactivates WNT pathway in HCC.

### Upregulation of miR-300 suppresses carcinogenesis in HCC

**Fig. 3:**
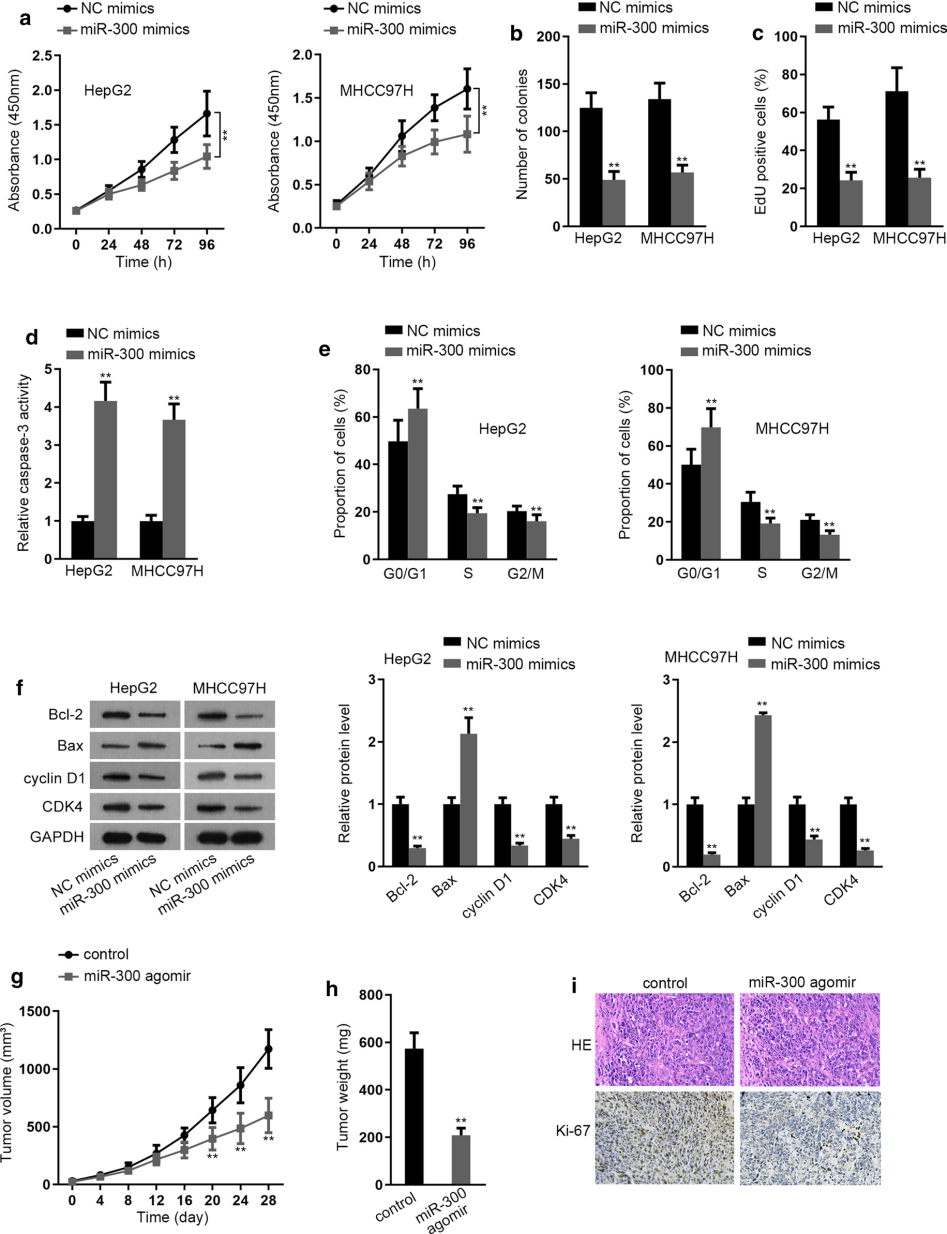
Upregulation of miR-300 suppressed tumorigenesis in HCC. **a**–**c** Cell proliferation ability was assessed in cells treated with miR-300 mimics or NC mimics by CCK-8, colony formation and EdU assays. **d** The effect of miR-300 upregulation on cell apoptosis was evaluated by caspase-3 activity test. **e** Cell cycle distribution was measured by flow cytometry analysis in HCC cells responding to the increased level of miR-300. **f** The proteins relevant to cell apoptosis and cell cycle were determined via western blot. **g**, **h** The growth curve and weight of tumors originated from MHCC97H cells transfected with control or miR-300 agomir. **i** The staining of Ki67 in above tumors was determined by IHC assay. **P < 0.01

Subsequently, the function of miR-300 in HCC were inquired. It manifested that the proliferation of two HCC cells was inhibited in response to miR-300 upregulation (Fig. 3a–c). However, the apoptosis was accelerated and cell cycle arrest was induced by increased miR-300 (Fig. 3d, e), which were further verified by the results of western blot (Fig. 3f). Additionally, the outcomes of in vivo experiments indicated the tumors derived from miR-300-enhanced cells were accompanied by slower growth rate, lighter weights and less Ki67 positivity than those from control cells (Fig. 3g–i). Therefore, we concluded that miR-300 inhibits HCC cell growth.

### LncRNA OIP5-AS1 sponges miR-300 and promotes cell growth in HCC

**Fig. 4:**
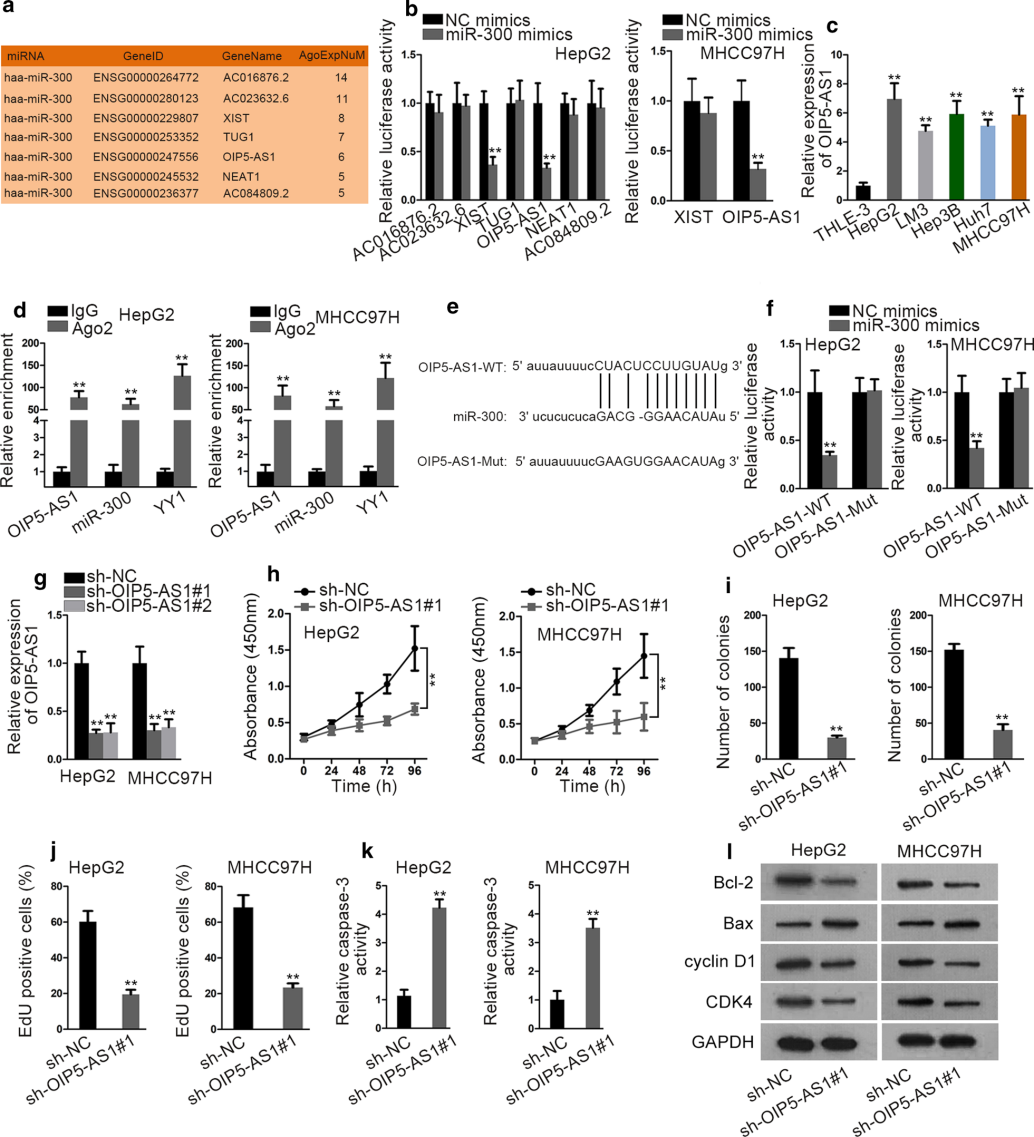
LncRNA OIP5-AS1 sponges miR-300 and promotes cell growth in HCC. **a** The upstream lncRNAs of miR-300 were predicted via starBase. **b** The impact of miR-300 on above predicted lncRNAs in HCC cells were detected by luciferase reporter assay. **c** OIP5-AS1 expression in five HCC cell lines and normal THLE-3 cells was detected by qRT-PCR. **d** The existence of miR-300, OIP5-AS1 and YY1 in RISCs was confirmed by RIP assay. **e** The binding site between miR-300 and OIP5-AS1 was predicted by starBase. **f** The interplay between miR-300 and OIP5-AS1 was examined by luciferase reporter assay. **g** OIP5-AS1 expression was examined by qRT-PCR after sh-NC or sh-OIP5-AS1#1/2 were transfected into HepG2 and MHCC97H cells. **h**–**j** The proliferation was assessed by CCK-8, colony formation and EdU assays in sh-OIP5-AS1#1 transfected HepG2 and MHCC97H cells. **k** Caspase-3 activity test and western blot analysis detected the influence of OIP5-AS1 silence on cell apoptosis and cell cycle in HepG2 and MHCC97H cells. **P < 0.01

To examine the ceRNA hypothesis, we wondered the upstream lncRNAs of miR-300. Through analyzing starBase, 7 potential lncRNAs were predicted to combine with miR-300 (Fig. 4a). As observed, co-transfection of miR-300 mimics led to decreased luciferase activity of OIP5-AS1 in both the two cells (Fig. 4b). Thus, we further focused on OIP5-AS1 in following experiments. It was unveiled that the expression of OIP5-AS1 was much higher in HCC cells than that in THLE-3 controls (Fig. 4c). Through RIP assay, we confirmed the strong enrichment of OIP5-AS1, miR-300 and YY1 in the magnetic beads conjugated with anti-Ago2 (Fig. 4d). Additionally, miR-300 was predicted by starBase to have binding sites on OIP5-AS1 (Fig. 4e). Importantly, the luciferase activity of OIP5-AS1-WT was markedly reduced by miR-300 overexpression, while that of OIP5-AS1-Mut showed no significant change (Fig. 4f). Further, the interplay between OIP5-AS1 and miR-300 was evidenced by RNA pull-down assay (Additional file 4: Fig. S3A). Then, to know the function of OIP5-AS1 in HCC, OIP5-AS1 was silenced for the loss-of-function assays (Fig. 4g), with no differences on the transfection efficiencies (Additional file 4: Fig. S3B). It was found that knocking down OIP5-AS1 significantly hampered the proliferative capacity of HCC cells (Fig. 4h–j). Additionally, silencing OIP5-AS1 induced cell apoptosis and cell cycle arrest in HCC (Fig. 4k, l and Additional file 4: Fig. S3C, D). Moreover, the retarding influence of silenced OIP5-AS1 on HCC tumor growth was further proven by in vivo animal study (Additional file 4: Fig. S3E–G). Taken together, OIP5-AS1 sponges miR-300 and promotes cell growth in HCC.

### YY1-mediated OIP5-AS1 promotes HCC cell growth through WNT pathway

**Fig. 5:**
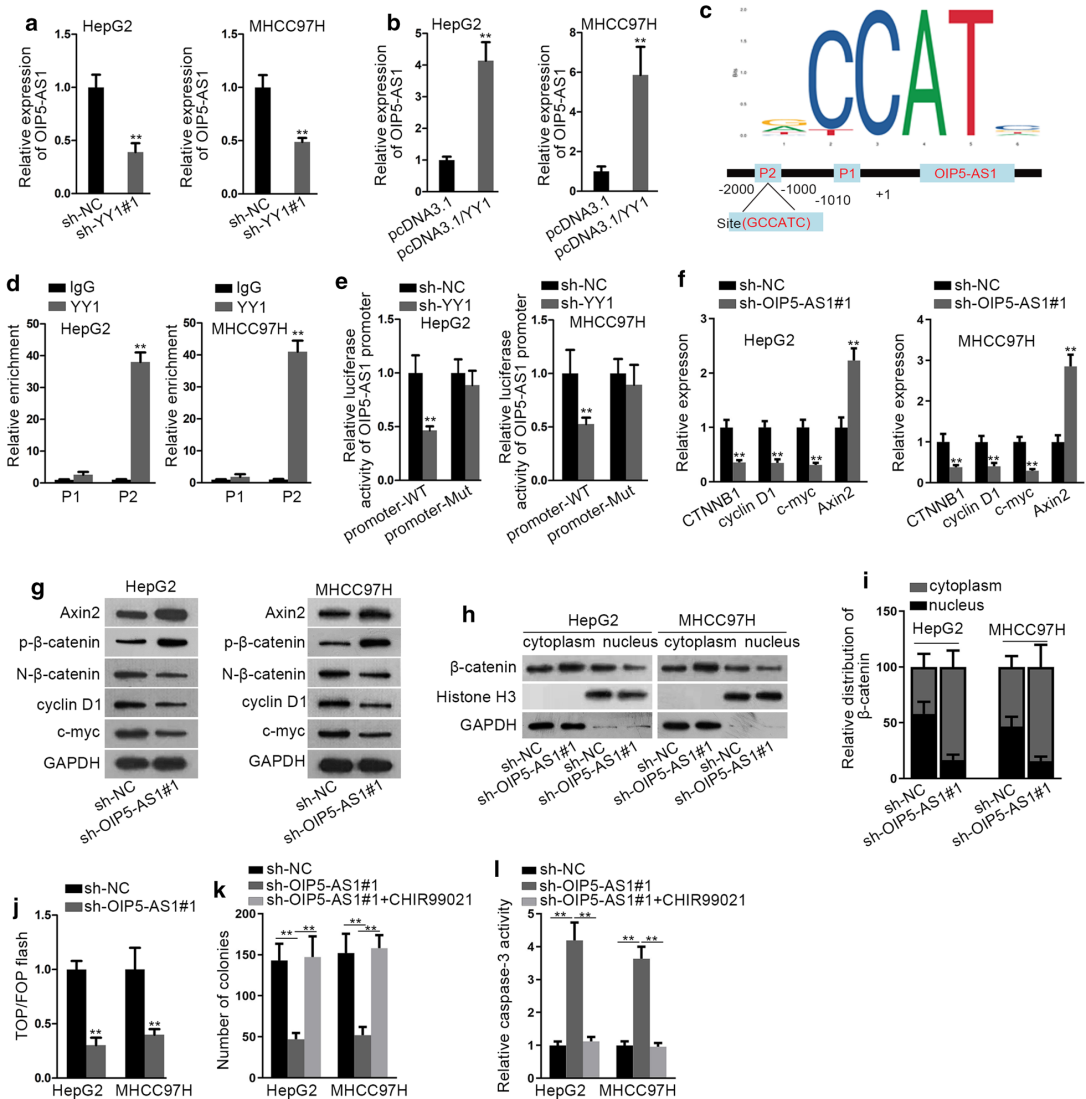
YY1-mediated OIP5-AS1 promotes HCC cell growth by activating WNT pathway. **a** Expression of OIP5-AS1 in YY1-silenced HCC cells was detected by qRT-PCR. **b** qRT-PCR was carried out to measure OIP5-AS1 expression in HCC cells upon YY1 upregulation. **c** The binding motif of YY1 and the binding site between YY1 and OIP5-AS1 promoter were predicted by JASPAR. **d** ChIP assay was conducted to determine the interaction between YY1 and OIP5-AS1 promoter. **e** The interaction between YY1 and OIP5-AS1 promoter was further verified by luciferase reporter assay. **f**, **g** The effect of OIP5-AS1 silencing on the mRNA and protein levels of Axin2, CTNNB1 (non-phosphorylated β-catenin and phosphorylated β-catenin), Cyclin D1 and c-myc was detected by qRT-PCR and western blot analyses. **h**, **i** The nuclear translocation of β-catenin was assessed by nuclear-cytoplasmic fractionation followed by western blot analysis in two OIP5-AS1-downregulated HCC cells. **j** The effect of OIP5-AS1 depletion on the activity of WNT pathway in HepG2 and MHCC97H cells was determined with the employment of TOP/FOP-flash luciferase reporter assay. **k** The proliferation of HCC cells transfected with sh-NC or sh-OIP5-AS1 when exposed to CHIR99021 was estimated by colony formation assay. **l** The apoptosis of indicated cells was examined by caspase-3 activity analysis. **P < 0.01

Interestingly, here we discovered that OIP5-AS1 expression was declined with YY1 downregulation but increased in YY1-overexpressed cells (Fig. 5a, b), implying that OIP5-AS1 was potentially regulated by YY1. It was commonly reported that YY1 functions as a transcription factor to activate the expressions of lncRNAs [29]. Hence, we hypothesized that YY1 might bind to OIP5-AS1 promoter to regulate OIP5-AS1 expression. Through UCSC (http://genome.ucsc.edu/) and JASPAR (http://jaspar.genereg.net/), YY1 was indicated as a probable transcription factor of OIP5-AS1. Then, the binding motif of YY1 and the binding sites of YY1 at OIP5-AS1 promoter were presented in Fig. 5c. To figure out the precise site for YY1 binding to OIP5-AS1 promoter, we fragmented the promoter to 2 parts, part 1 (P1: − 1010 ~ +1) and part 2 (P2: − 2000 ~ − 1000). Intriguingly, the results of ChIP assay displayed that only P2 of OIP5-AS1 promoter was harvested by anti-YY1 (Fig. 5D), indicating that YY1 interacted with OIP5-AS1 promoter in P2. Further, we unmasked that YY1 silencing considerably decreased the luciferase activity of wild type OIP5-AS1 promoter, without any influences on that of the mutant promoter (Fig. 5e). Thereafter, we probed into the effect of OIP5-AS1 on WNT pathway. As expected, the levels of CTNNB1, cyclin D1 and c-myc were notably reduced while the expression of Axin2 elevated under OIP5-AS1 silence (Fig. 5f). In addition, the protein levels of non-phosphorylated β-catenin, cyclin D1 and c-myc were reduced but that of Axin2 and phosphorylated β-catenin presented an opposite tendency (Fig. 5g and Additional file 5: Fig. S4A). Then, silenced OIP5-AS1 was confirmed to hamper the translocation of β-catenin via nuclear-cytoplasmic fractionation followed by western blot analysis (Fig. 5h, i), which was further validated by IF assays (Additional file 5: Fig. S4B). Subsequently, downregulated OIP5-AS1 considerably lessened TOP activity (Fig. 5j). Importantly, the aggregation rate of these two HCC cells was decreased a lot in response to the silence of OIP5-AS1 (Additional file 5: Fig. S4C). Furthermore, TOP/FOP flash examination indicated that the decreased luciferase activity of TOP flash caused by miR-300 mimics was gradually recovered by the overexpression of OIP5-AS1 (Additional file 5: Fig. S4D). These data uncovered OIP5-AS1 participated in activating WNT pathway. CHIR99021, the WNT signaling activator, was subsequently used for rescue assays. As shown in Fig. 5k, the treatment of CHIR99021 abrogated the suppression of OIP5-AS1 deficiency on cell proliferation. Moreover, we demonstrated that OIP5-AS1 inhibition-stimulated cell apoptosis was subsequently counteracted by CHIR99021 (Fig. 5l). To be concluded, YY1-mediated OIP5-AS1 promotes HCC cell growth by activation of WNT pathway.

### OIP5-AS1 facilitates HCC cell growth by modulating YY1

**Fig. 6:**
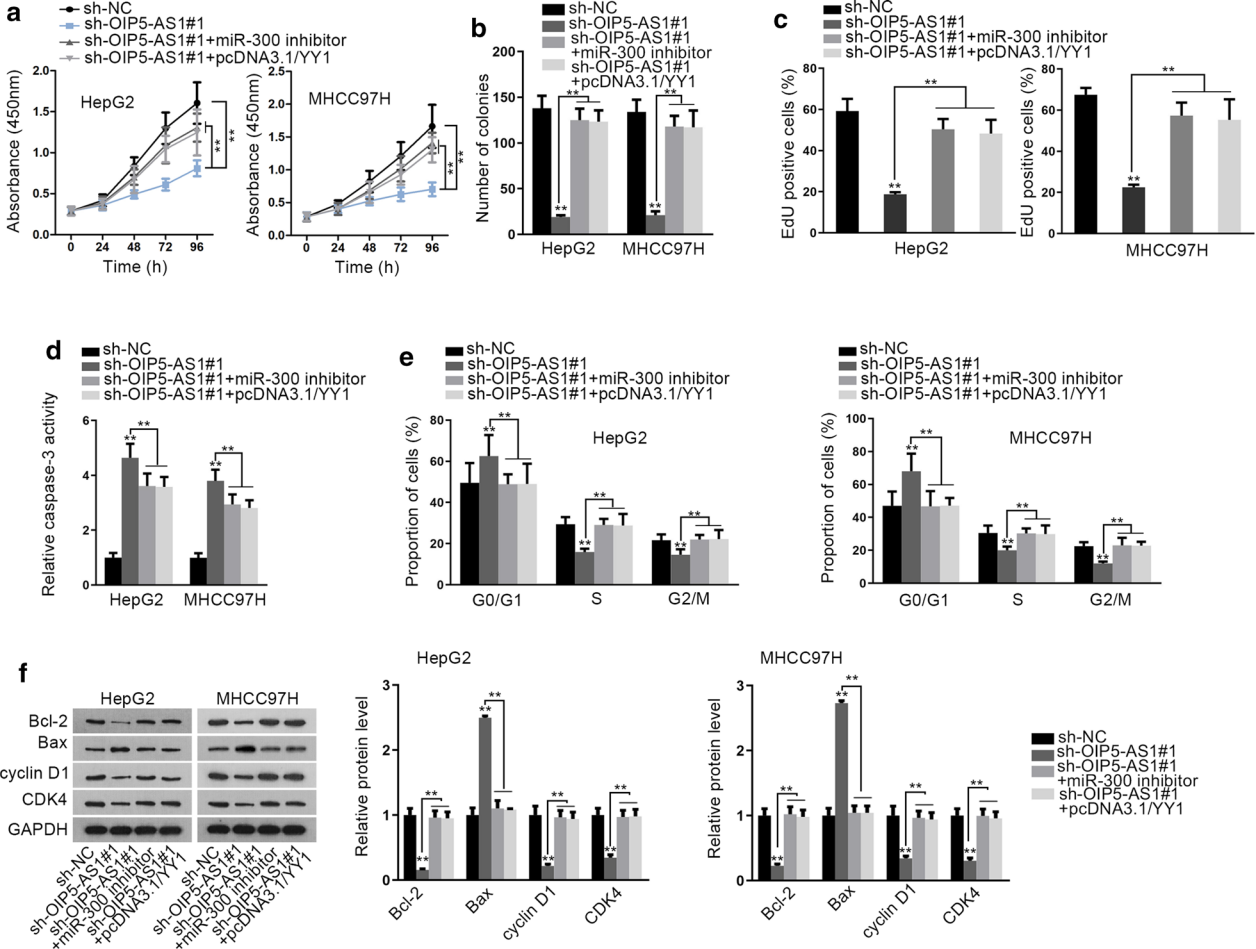
OIP5-AS1 facilitates HCC cell growth by modulating miR-300/YY1 axis. **a**–**c** The proliferation of HCC cells in these groups (sh-NC, sh-OIP5-AS1, sh-OIP5-AS1 + miR-300 inhibitor, or sh-OIP5-AS1 + pcDNA3.1/YY1) was estimated by CCK-8, colony formation and EdU assays. **d**, **e** The apoptosis and cell cycle distribution of HCC cells under above contexts were tested by caspase-3 activity test and flow cytometry analysis. **f** Western blot analyzed the apoptosis and cell cycle related proteins in HCC cells in response to above conditions. **P < 0.01

To further study the regulation of OIP5-AS1/miR-300/YY1 feedback loop in HCC cell growth, we conducted following rescue assays. It was discovered that upregulated YY1 or downregulated miR-300 restored the inhibition of OIP5-AS1 depletion on the proliferation of HCC cells (Fig. 6a–c). Subsequently, results from caspase-3 activity assay indicated that the deficiency of OIP5-AS1 dramatically repressed cell apoptosis in HCC cells, while the co-transfection of pcDNA3.1/YY1 or miR-300 inhibitor abolished above effect (Fig. 6d). Moreover, the cell cycle arresting impact of depleted OIP5-AS1 was also offset in face of miR-300 inhibition or YY1 upregulation (Fig. 6e). Similarly, the influences of OIP5-AS1 silence on the levels of proteins related to cell apoptosis and cell cycle were countervailed by suppressed miR-300 or enhanced YY1 (Fig. 6f) In summary, OIP5-AS1 facilitates HCC cell growth by modulating miR-300/YY1 signaling.

## Discussion

In recent years, the pre-clinical treatment strategies have been gradually reported [3, 4]. Despite great improvement in interventional therapy has been made for HCC, the clinical effect remains disappointed due to the difficulty in early diagnosis of HCC patients [5]. This study explored the molecular mechanism underlying the HCC tumor growth, thereby providing new possible therapeutic targets for HCC.

Yin-Yang 1 (YY1) was identified as a pivotal regulator for diverse diseases. As an example, YY1 restrains pancreatic ductal adenocarcinoma cell proliferation and migration through transcriptionally activating CDKN3 expression [30]. Through proteomic analysis, YY1 was acknowledged as a regulator in prostate cancer [31]. Furthermore, YY1 contributes to the tumorigenesis and progression in laryngeal cancer by directly regulating MYCT1 [32]. In our study, YY1 was upregulated in HCC cells. Upregulated YY1 facilitated the proliferation but inhibited the apoptosis in HCC cells. Besides, YY1 was validated to be a growth promoter in HCC. In brief, YY1 was identified as an oncogene in HCC.

MicroRNAs (miRNAs), with about 24 nucleotides, are small RNAs that lack ability related to code proteins [33]. It was recognized that miRNAs could be complementally paired with the 3′-UTR of mRNA and thereby promoted mRNA degradation or inhibited its translation [34]. Accumulating studies have reported the regulatory role of miRNAs in tumor growth [35]. MiRNA-518 hinders cell proliferation and stimulates apoptosis by modulating MDM2 in gastric cancer [36]. Elevated miR-183 contributes to the radioresistance of glioblastoma through decreasing LRIG1 expression [37]. Up to now, increasing reports uncovered that miRNAs play pivotal parts in HCC development [38–40]. Low-expressed miR-138 and miR-1271 restrains metastasis and EMT process of HCC cells [41, 42]. In addition, miR-1468 promotes HCC tumor growth via acting as an oncogene [43]. MiR-300 has been discovered in numerous diseases, for instance, osteosarcoma [44], pancreatic cancer [28] and knee osteoarthritis [45]. Here, we unveiled that miR-300 expression was observably downregulated in HCC cells. Furthermore, miR-300 targeted to YY1 and negatively modulated the expression level of YY1. Finally, it was discovered that miR-300 associated with WNT pathway.

In recent decades, it has been confirmed that dysregulation of lncRNAs is closely connected with tumor growth of human cancers. Former researches have revealed the involvement of lncRNAs in hepatocellular carcinoma (HCC). Through regulating ERBB2IP, lncRNA KTN1-AS1 serves as an oncogenic facilitator in HCC [46]. By targeting miR-217/MAPK1 axis, lncRNA CRNDE promotes the malignancy in HCC [47]. The dysregulation of WNT signaling has been disclosed in many diseases [48]. WNT pathway inhibited by LASP2 was to promote malignancy in bladder cancer [49]. And the regulation of WNT signaling was also researched in HCC. It was reported that HDAC6 inhibits WNT pathway to suppress cell proliferation in HCC [50]. Herein, we unmasked that lncRNA OIP5-AS1 targeted to miR-300 and enhanced HCC cell growth. Further, it was fund that OIP5-AS1 was activated by YY1 and promoted cell growth by activating WNT pathway in HCC. WNT signaling pathway is complicated and developing, which could contribute to the progression of human cancers. Except for canonical WNT pathway, WNT/Planar Cell Polarity (PCP) pathway has also been studied in recent years due to its role in controlling cancer cell functions [51–53]. Current study determined the activation of OIP5-AS1/miR-300/YY1 feedback loop on canonical WNT pathway. However, whether OIP5-AS1 could regulate WNT/PCP pathway is still a secret, which is also a limitation of our current study. Thus, we will focus on the association between OIP5-AS1 and WNT/PCP pathway in our future study and detect more biological functions of OIP5-AS1/miR-300/YY1 axis in HCC.

## Conclusion

In conclusion, our results delineated that OIP5-AS1/miR-300/YY1 feedback loop facilitates cell growth in HCC by activating WNT canonical pathway, which might helpful for searching new treatments for HCC.

## Supplementary information

**Additional file 1.** Additional file.

**Additional file 2: Figure S1.** The effects of overexpressed OIP5-AS1 on the functions of normal hepatocytes. (A) Transfection efficiency of sh-NC or sh-YY1#1/2 in HepG2 and MHCC97H cells was detected by flow cytometry analysis. (B) The quantification of protein bands detected by western blot of Fig. 1h. (C) The transfection efficiency of pcDNA3.1 or pcDNA3.1-YY1 in these two HCC cells was determined by flow cytometry analysis. (D) YY1 expression in HCC cells transfected with pcDNA3.1 or pcDNA3.1-YY1 was evaluated by qRT-PCR. (E-J) Gain-of function assays were conducted in THLE-3 cells, including CCK-8 (E), colony formation (F), EdU (G), caspase-3 activity test (H), flow cytometry analysis (I) and western blot analysis (J). **P < 0.01. n.s. indicated data were not statistically significant.

**Additional file 3: Figure S2.** The effect of YY1 or miR-300 on WNT pathway. (A) Luciferase reporter assay identified the role of YY1 in regulating the transcription of WNT-related factors. (B) Transfection efficiency of increasing dose of pcDNA3.1-YY1 was measured via flow cytometry analysis. (C) YY1 expression under above transfections was determined by qRT-PCR. (D) TOP/FOP flash assay assessed the activity of WNT pathway in response to increasing YY1 expression. (E) The quantification of bands in blots of Fig. 2e. (F) The nuclear translocation of β-catenin in HCC cells with or without miR-300 upregulation was detected by IF analysis. *P < 0.05, **P < 0.01. n.s. indicated data were not statistically significant.

**Additional file 4: Figure S3.** OIP5-AS1 affected HCC cell growth both in vitro and in vivo. (A) RNA pull-down assay detected the interaction between miR-300 and OIP5-AS1. (B) Transfection efficiency of OIP5-AS1-specfic shRNAs was measured by flow cytometry analysis. (C) Cell cycle distribution in HCC cells with or without OIP5-AS1 silence was measured by flow cytometry analysis. (D) Quantification of protein bands shown in Fig. 4l. (E-F) The growth curve and weight of tumors in mice injected with sh-NC or sh-OIP5-AS1#1-transfected MHCC97H cells. (G) IHC assay analyzed the Ki67 expression in tumors from above two groups. **P < 0.01.

**Additional file 5: Figure S4.** OIP5-AS1 silence inactivated WNT signaling. (A) Quantification of protein bands shown in Fig. 5g. (B) IF assay indicated that depletion of OIP5-AS1 prevented β-catenin translocating into nucleus. (C) The aggregation rate of two HCC cells after silence of OIP5-AS1 was measured via cell aggregation assay. (D) TOP/FOP flash examination indicated that the decreased luciferase activity of TOP flash caused by miR-300 mimics was gradually recovered by the overexpression of OIP5-AS1. **P < 0.01.

## Data Availability

Research data and material are not shared.

## Abbreviations

lncRNAs: Long non-coding RNAs
ceRNA: Competing endogenous RNA
OIP5-AS1: OIP5 antisense RNA 1
miRNAs: microRNAs
HCC: Hepatocellular carcinoma
mRNA: Messenger RNA
FBS: Fetal bovine serum
qRT-PCR: Quantitative real-time polymerase chain reaction
GAPDH: Glyceraldehyde-3-phosphate dehydrogenase
shRNA: Short hairpin RNA
NC: Negative control
CCK-8: Cell counting kit-8
PBS: Phosphate buffer saline
BCA: Bicinchoninic acid
PVDF: Polyvinylidene fluoride
ChIP: Chromatin immunoprecipitation
SD: Standard deviation
YY1: Yin-Yang 1
