# Supplementary figures and images for: OIP5-AS1 contributes to tumorigenesis in hepatocellular carcinoma by miR-300/YY1-activated WNT pathway
Source: Cancer Cell Int. 2020 Sep 9;20:440. doi: 10.1186/s12935-020-01467-6 (PMC7487829; doi:10.1186/s12935-020-01467-6)

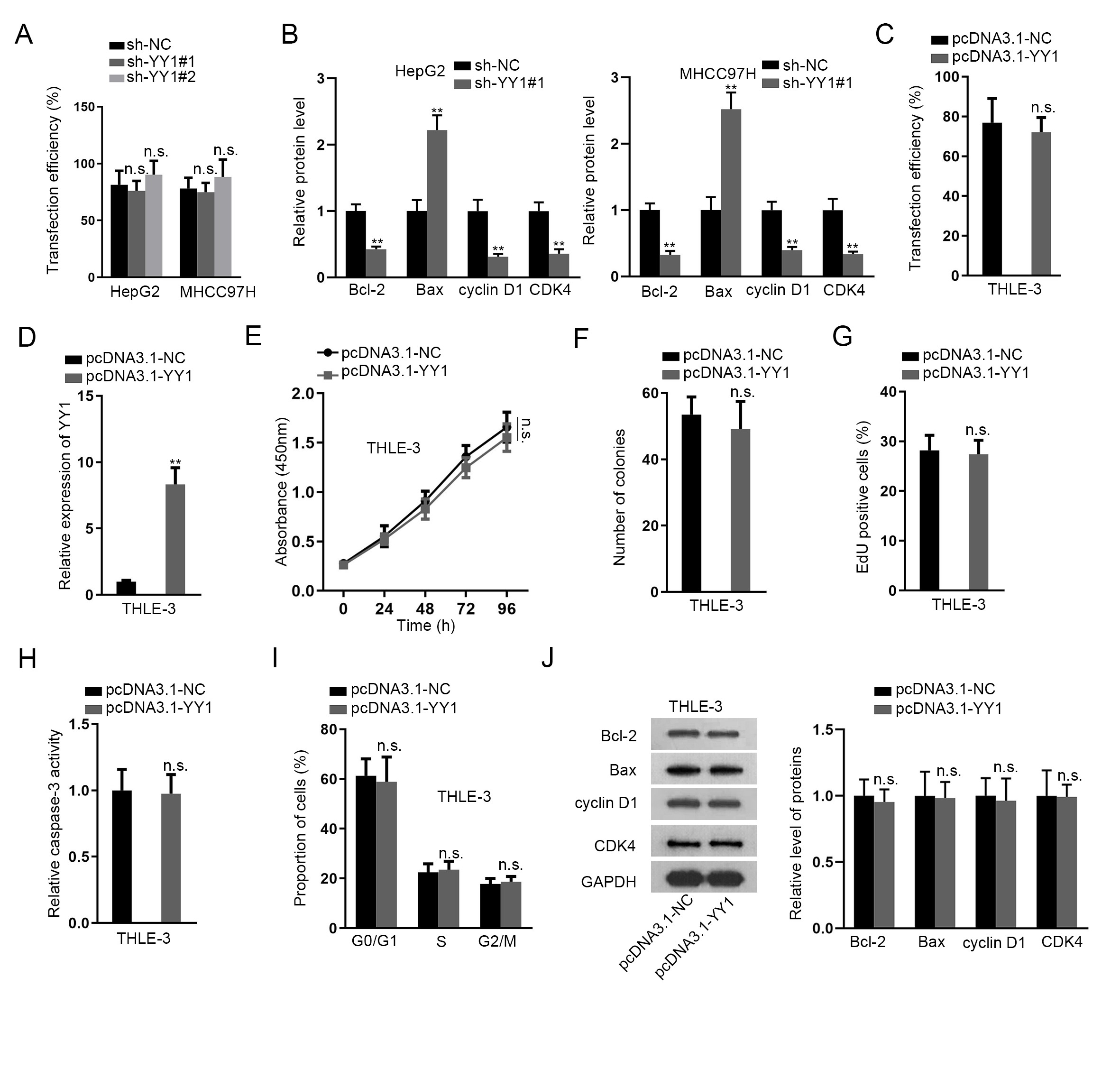

Supplement: Supplementary file 2 — Additional file 2: Figure S1. The effects of overexpressed OIP5-AS1 on the functions of normal hepatocytes. (A) Transfection efficiency of sh-NC or sh-YY1#1/2 in HepG2 and MHCC97H cells was detected by flow cytometry analysis. (B) The quantification of protein bands detected by western blot of Fig. 1h. (C) The transfection efficiency of pcDNA3.1 or pcDNA3.1-YY1 in these two HCC cells was determined by flow cytometry analysis. (D) YY1 expression in HCC cells transfected with pcDNA3.1 or pcDNA3.1-YY1 was evaluated by qRT-PCR. (E-J) Gain-of function assays were conducted in THLE-3 cells, including CCK-8 (E), colony formation (F), EdU (G), caspase-3 activity test (H), flow cytometry analysis (I) and western blot analysis (J). **P < 0.01. n.s. indicated data were not statistically significant. [file 12935_2020_1467_MOESM2_ESM.tif]

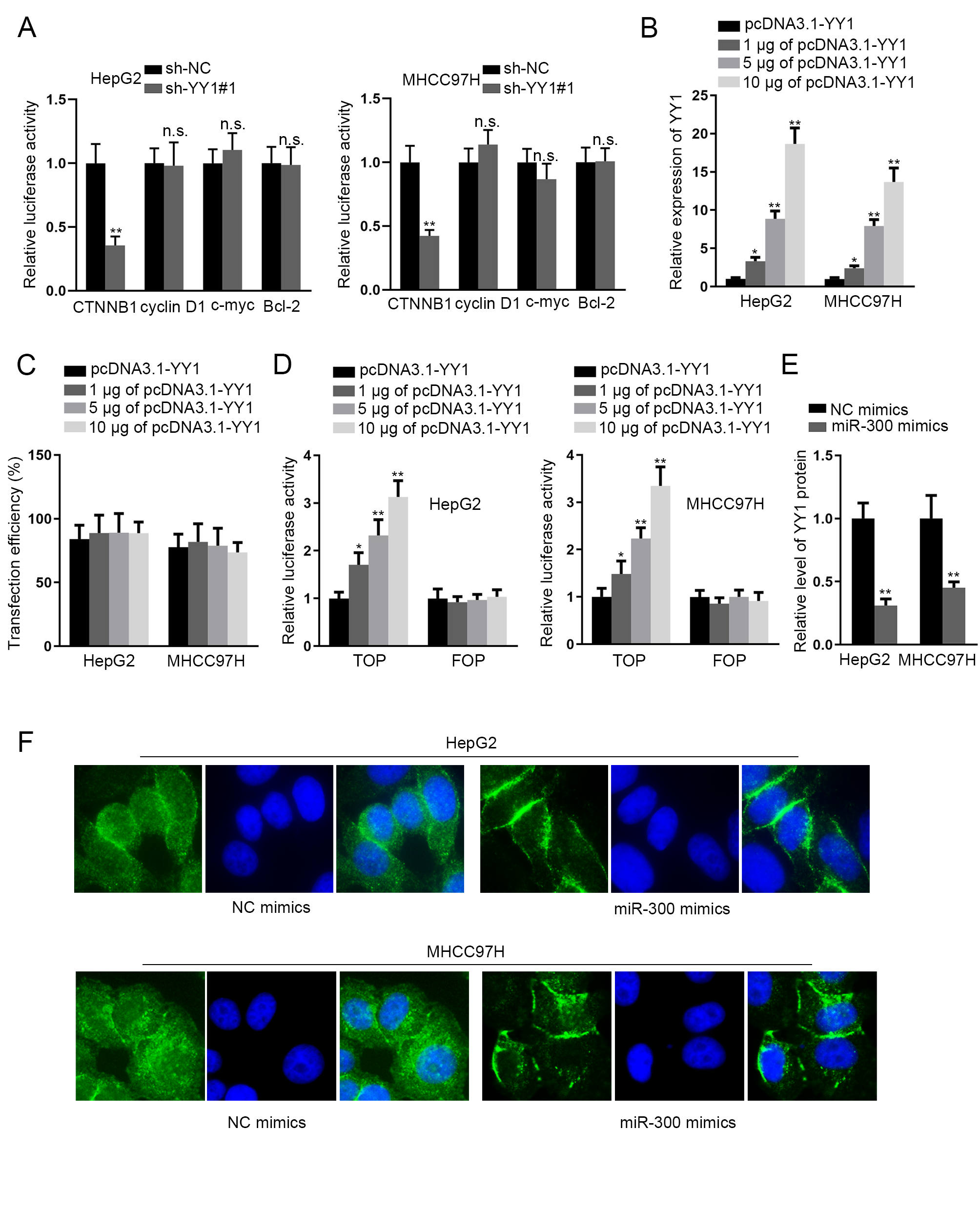

Supplement: Supplementary file 3 — Additional file 3: Figure S2. The effect of YY1 or miR-300 on WNT pathway. (A) Luciferase reporter assay identified the role of YY1 in regulating the transcription of WNT-related factors. (B) Transfection efficiency of increasing dose of pcDNA3.1-YY1 was measured via flow cytometry analysis. (C) YY1 expression under above transfections was determined by qRT-PCR. (D) TOP/FOP flash assay assessed the activity of WNT pathway in response to increasing YY1 expression. (E) The quantification of bands in blots of Fig. 2e. (F) The nuclear translocation of β-catenin in HCC cells with or without miR-300 upregulation was detected by IF analysis. *P < 0.05, **P < 0.01. n.s. indicated data were not statistically significant. [file 12935_2020_1467_MOESM3_ESM.tif]

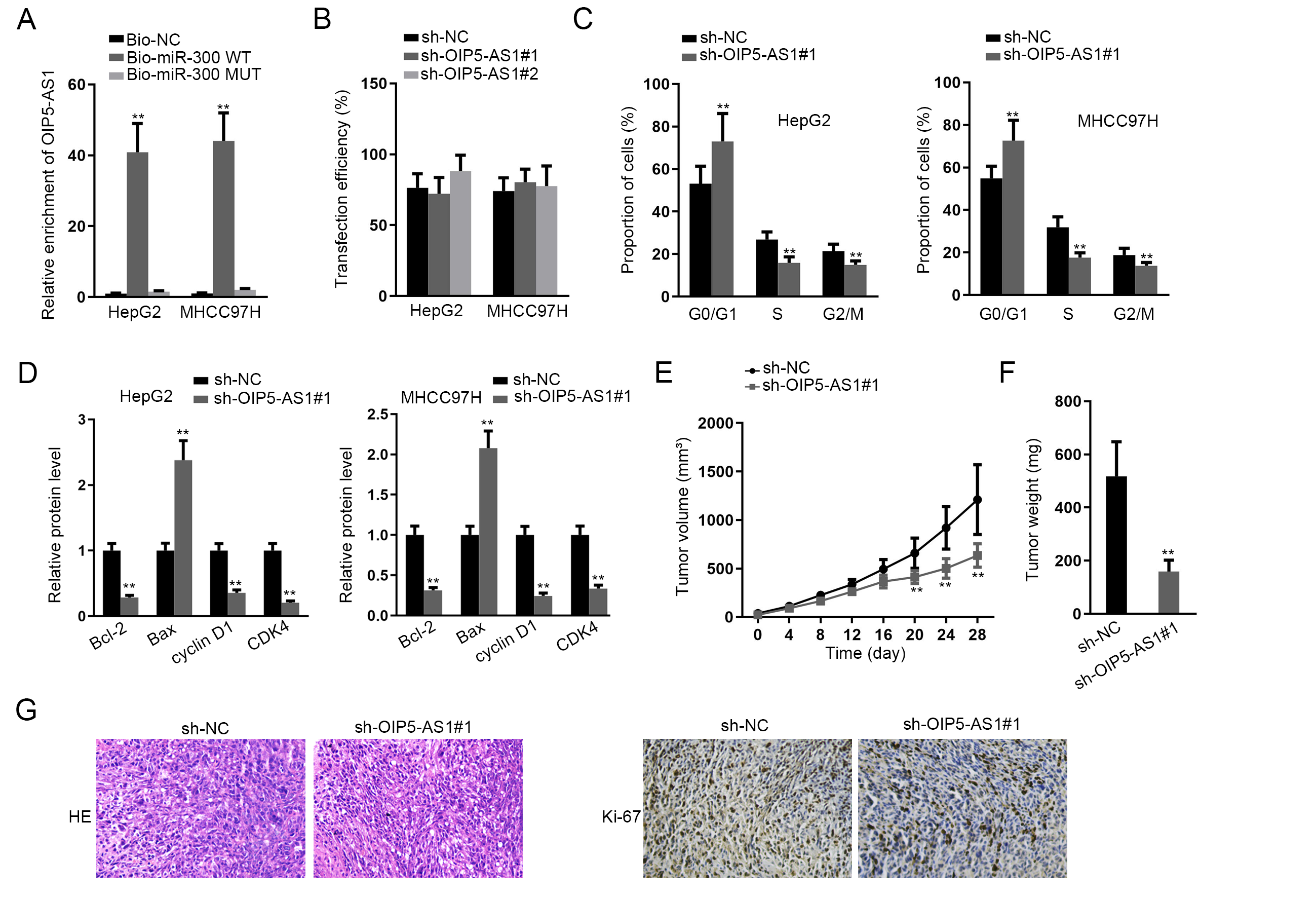

Supplement: Supplementary file 4 — Additional file 4: Figure S3. OIP5-AS1 affected HCC cell growth both in vitro and in vivo. (A) RNA pull-down assay detected the interaction between miR-300 and OIP5-AS1. (B) Transfection efficiency of OIP5-AS1-specfic shRNAs was measured by flow cytometry analysis. (C) Cell cycle distribution in HCC cells with or without OIP5-AS1 silence was measured by flow cytometry analysis. (D) Quantification of protein bands shown in Fig. 4l. (E-F) The growth curve and weight of tumors in mice injected with sh-NC or sh-OIP5-AS1#1-transfected MHCC97H cells. (G) IHC assay analyzed the Ki67 expression in tumors from above two groups. **P < 0.01. [file 12935_2020_1467_MOESM4_ESM.tif]

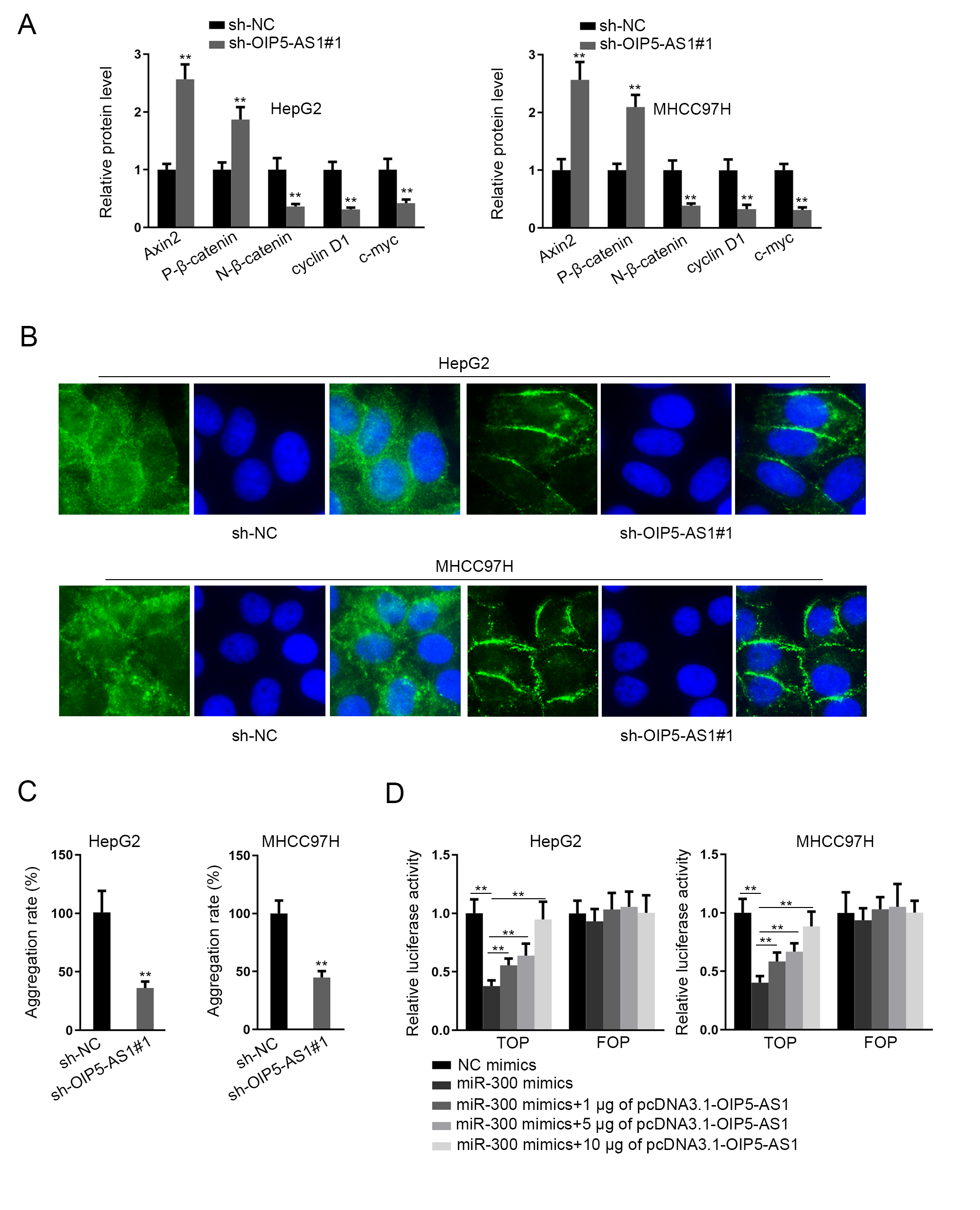

Supplement: Supplementary file 5 — Additional file 5: Figure S4. OIP5-AS1 silence inactivated WNT signaling. (A) Quantification of protein bands shown in Fig. 5g. (B) IF assay indicated that depletion of OIP5-AS1 prevented β-catenin translocating into nucleus. (C) The aggregation rate of two HCC cells after silence of OIP5-AS1 was measured via cell aggregation assay. (D) TOP/FOP flash examination indicated that the decreased luciferase activity of TOP flash caused by miR-300 mimics was gradually recovered by the overexpression of OIP5-AS1. **P < 0.01. [file 12935_2020_1467_MOESM5_ESM.tif]
